# Gender-based analysis of factors affecting junior medical students’ career selection: addressing the shortage of surgical workforce in Rwanda

**DOI:** 10.1186/s12960-018-0295-7

**Published:** 2018-07-11

**Authors:** Grace Kansayisa, Sojung Yi, Yihan Lin, Ainhoa Costas-Chavarri

**Affiliations:** 1 Department of Surgery, Centre Hospitalier Universitaire de Kigali, Kigali, Rwanda; 2 Program in Global Surgery and Social Change, Harvard Medical School, Boston, MA United States of America; 3 Department of Surgery, University of Colorado Hospital, Denver, CO United States of America; 4 Department of Surgery, Rwanda Military Hospital, Kigali, Rwanda

**Keywords:** Global health, Surgery, Medical education, Rwanda, Eastern Africa

## Abstract

**Background:**

There is a strong need for expanding surgical workforce in low- and middle-income countries. However, the number of medical students selecting surgical careers is not sufficient to meet this need. In Rwanda, there is an additional gender gap in speciality selection. Our study aims to understand the early variables involved in junior medical students’ preference of specialisation with a focus on gender disparities.

**Methods:**

We performed a cross-sectional survey of medical students during their clinical rotation years at the University of Rwanda. Demographics, specialisation preference, and factors involved in that preference were obtained using questionnaires and analysed using descriptive statistics and odds ratios.

**Results:**

One hundred eighty-one respondents participated in the study (49.2% response rate) with a female-to-male ratio of 1 to 2.5. Surgery was the preferred speciality for 46.9% of male participants, and obstetrics/gynaecology for 29.4% of females. The main selection criteria for those who had already decided on surgery as a career included intellectual challenge (60.0%), interaction with residents (52.7%), and core clerkship experience (41.8%) for male participants and interaction with residents (57.1%), intellectual challenge (52.4%), and core clerkship experience (52.4%) for female participants. Females were more likely than males to join surgery based on perceived research opportunities (OR 2.7, *p* = 0.04). Male participants were more likely than their female participants to drop selection of surgery as a speciality when an adverse interaction with a resident was encountered (OR 0.26, *p* = 0.03).

**Conclusion:**

This study provides insight into factors that guide Rwandan junior medical students’ speciality preference. Medical students are more likely to consider surgical careers when exposed to positive clerkship experiences that provide intellectual challenges, as well as focused mentorship that facilitates effective research opportunities. Ultimately, creating a comprehensive curriculum that supports students’ preferences may help encourage their selection of surgical careers.

## Background

Rwanda is a densely populated country in East Africa, with over 67% of residents aged less than 20 years [1]. The Rwandan health system was devastated after the genocide of 1994, when nearly 20% of the population was killed and innumerable others left with permanent disabilities [2]. Tremendous progress has been achieved since to rebuild and expand the health care system. However, the health sector is still in significant need of resources to address the surgical burden: surgeons and other surgical personnel, as well as equipment and infrastructure [3].

Rwanda has approximately 709 physicians, most of whom are general practitioners. These general practitioners provide the majority of surgical care, with 82% of general surgery and obstetric procedures performed at district hospitals [4]. There are approximately 50 fully trained surgeons, which translates to a ratio of 0.49 total surgeons per 100 000 population. In contrast, the Lancet Global Surgery Commission recommends a country’s minimum surgical physicians-to-population ratio to be 20 per 100 000 [5].

A closer look at the number of medical students and residents at the University of Rwanda is helpful in anticipating workforce trends. There are currently 90–100 medical students per class, and approximately 33 residency positions in surgery and surgical sub-specialities available each year: 10 in general surgery, 13 in obstetrics/gynaecology, five in orthopaedics, three in urology, and two in neurosurgery. There are also approximately 12 positions available in anaesthesia each year. The majority of surgical residents are male, with an average female-to-male ratio of 1 to 15.5. This female-to-male ratio is disproportionate to the average 1 to 2.3 ratio in the overall medical student population.

In Rwanda, the medical training system is set up in such a way that clinical rotations at district hospitals provide limited exposure to general surgical cases. It may be common for a medical student to not receive sufficient exposure to surgical cases during the final sixth year prior to choosing a speciality. Furthermore, there are few female surgeons who can serve as examples and mentors for female medical students to pursue a career in surgery. The lack of mentorship, or even poor quality of existing limited mentorship, may compromise the medical students’ perception of a surgical career. Indeed, mentorship has been shown to be important in previous studies demonstrating that role model and personality traits were significant factors influencing selection of surgery [6].

Various studies from other African countries offer different perspectives on unique factors involved in selecting surgical careers. In Nigeria, a majority of students expressed concern for the increased risk of HIV/AIDS associated with surgical specialities, although it ultimately did not have a statistically significant effect on speciality selection [7]. In Kenya, a descriptive cross-sectional study showed that a preference for urban practice setting contributed to students’ consideration of surgical specialisation [8]. These factors involved in the process of selecting a speciality suggest that there may be variables that are unique to Rwanda too.

However, there are no previous studies attempting to identify the many factors involved in Rwandan medical students’ decision process of selecting specialisation. A better understanding of these factors may help encourage more students to enter a surgical specialisation and address the need for strengthening the Rwandan surgical workforce.

Junior medical students were targeted for this study because the speciality selection process begins in earnest during clinical rotations when students are exposed to the clinical and intellectual environments of the various specialities [9]. This period is significant for making initial impressions about the practical aspects of medical practice and the various specialities. Gaining insight into the junior medical students’ most important criteria for selecting medical specialities during this period may provide better understanding of that early decision process.

This study will examine factors involved in junior medical students’ selection of a surgical career in Rwanda. Understanding these factors may help recognise the various barriers that students face, and ultimately aid in identifying ways by which more students will be encouraged to enter a career in surgery.

## Methods

### Study setting

The study was conducted in the College of Medicine and Health Sciences at the University of Rwanda, the largest public university in the country. There are approximately 600 students in the medical school. The medical school has separate locations depending on the training year: the junior classes (year 1 through 3) are held in Huye in the southern province of the country, while senior classes (year 4 through 5) have classes in the capital city of Kigali. This geographically widespread campus increases students’ clinical exposure by providing them access to more than just the three referral hospitals in the country (Kigali University Teaching Hospital, Butare University and Teaching Hospital, and Rwanda Military Hospital). Year 6 students are further widespread geographically, as personal interest may dictate preference for clinical setting and patient population. Clinical exposure begins in year 3 when students attend their clerkships through the four core clinical departments (internal medicine, obstetrics and gynaecology, paediatrics, and surgery). Students repeat these core rotations in year 4, so they do not rotate in the other required specialities, including anaesthesia, or other surgical sub-specialities until year 5. As such, most junior medical students are considering and deciding between the four core specialities, unless they have been exposed to other clinical experiences prior to or in parallel with this standardised training curriculum in Rwanda.

### Study design

This is a cross-sectional study surveying medical students currently enrolled at the University of Rwanda in their first two clinical years (years 3 and 4). The survey collects demographic details (i.e. age, sex, marital status) and answers to both open and binary yes/no questions to evaluate interest and factors involved in selecting a speciality. The survey was administered in the beginning months of the medical students’ academic year.

The survey queries the following: demographic details, clerkship exposure so far, first preference speciality, when and where that preference was determined, factors affecting that preference, preference speciality in an ideal context, and, again, factors affecting that ideal preference. In recognition that many students would change their preference of specialisation if there were more control over certain variables such as age, length of training, family responsibilities, financial limitations, academic achievement, etc., and given that this survey intended to elucidate such variables specific to Rwandan trainees, an “ideal” context was offered to observe any changes in reported preferences. Moreover, open response sections were provided in addition to a list of possible variables affecting speciality preference, such that participants could clarify their decision-making process.

### Data analysis

The results were analysed with Stata 14.2 using descriptive statistics to identify characteristics of students and their specialisation factors. Odds ratios were calculated to compare each result by gender. Significance was determined as *p* < 0.05.

### Ethical approval

Ethical approval for this study was sought and obtained from the University of Rwanda Institutional Review Board (No. 362/CMHS IRB/2016).

## Results

### Demographics

A total of 181 questionnaires were returned, for a response rate of 49.2%. Except where stated, data from all 181 questionnaires were included in the data analysis.

Male-to-female ratio of students was approximately 1:2.5 (Table 1). The mean age for both female and male students (*n* = 181) was 23 years with a range of 20 to 38 years. Both genders were evenly distributed within the sample size. There was also no significant difference in the marital status of both male and female participants.

**Table 1:** Participant demographics

	Male	Female	*p* value
Age (mean years)	23.13	22.5	0.15
Marital status			
Single	95.4%	94.1%	0.71
Not single	4.6%	5.9%	

### First preference specialities by gender

Participants reported their current first preferences for speciality (Table 2). Surgery was selected most commonly at 46.9% amongst males, while obstetrics and gynaecology was selected most commonly at 29.4% amongst females. Internal medicine was the second most chosen speciality for males at 24.6%, while surgery was the second most chosen for females at 19.6%.

**Table 2:** First preference specialities, distributed by gender

	Male	Female	Total	Odds ratio	*p* value
	%	%	*n*		
Surgery	46.9	19.6	71	0.31	< 0.01
Obstetrics/gynaecology	18.5	29.4	39	2.14	0.05
Internal medicine	24.6	11.8	38	0.46	0.11
Paediatrics	5.4	15.7	15	3.7	0.02
Other	4.6	13.7	13	3.71	0.03

Females were significantly less likely than their male counterparts to choose surgery as their first option (OR 0.31, *p* < 0.01) (Table 2). However, females were twice as likely to select obstetrics and gynaecology compared to males (OR 2.14, *p* = 0.05), and almost four times more likely to select paediatrics (OR 3.7, *p* = 0.02) or any other speciality other than surgery (OR 3.7, *p* = 0.03). There was no significant difference in choosing internal medicine between genders (male 24.6% vs. female 11.8%, OR 0.46, *p* = 0.11).

### Preference of specialities in an ideal context by gender

When asked about which speciality would be preferred under ideal circumstances where all variables could be controlled, both male and female participants provided an almost similar distribution of responses (Table 3). In an ideal context, males opting for surgery changed from 46.9% (Table 2) to 42.3% (Table 3), while females choosing surgery doubled from 19.6% (Table 2) to 41.2% (Table 3). Female participants chose internal medicine at the lowest rate (1.96%). Males were more likely to choose internal medicine compared to females in an ideal context where they could control all other variables in their speciality selection (OR 0.2, *p* = 0.02).

**Table 3:** Preference of specialities in an ideal context, distributed by gender

	Male	Female	Total	Odds ratio	*p* value
	%	%	*n*		
Surgery	42.3	41.2	76	0.965	0.923
Internal medicine	21.54	1.96	31	0.217	0.02
Obstetrics/gynaecology	10.77	19.61	24	2.12	0.106
Paediatrics	1.54	3.92	4	2.66	0.337
Other	2.31	7.84	6	2.75	0.168

### Factors affecting choice of specialisation

**Fig. 1:**
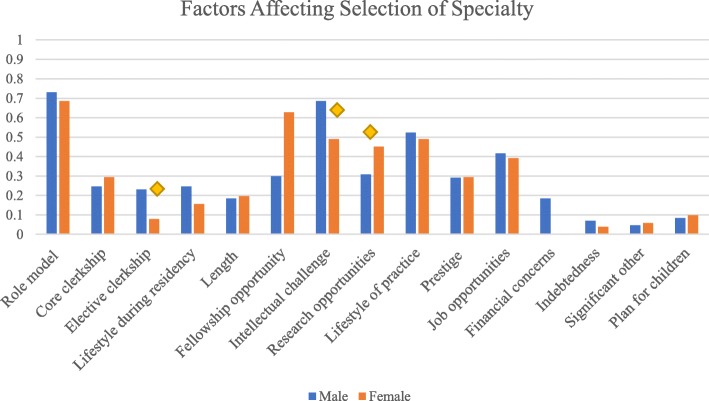
Factors affecting selection of speciality. Diamond indicates *p <* 0.05

In discussing factors affecting selection of speciality (Fig. 1), the most common three reasons cited by male participants included having a role model (73.1%), feeling intellectual challenge (69.5%), and considering lifestyle of practice (24.6%). For females, the top factors were having a role model (68.6%), potential fellowship opportunities (62.7%), feeling intellectual challenge (49.0%), and considering lifestyle of practice (49.0%). Males were more likely than females to be drawn to choose a speciality based on the intellectual challenge factor (OR 0.48, *p* = 0.03), as well as elective experience within a specific speciality (OR 0.28, *p* = 0.02). Alternatively, females were more prone than their male colleagues to choose their speciality when considering “research opportunity” (OR 1.99, *p* = 0.04). There was no statistical difference by gender for citing prestige and length of study as factors impacting specialisation choice.

### Factors influencing choice of surgery as a specialisation

**Fig. 2:**
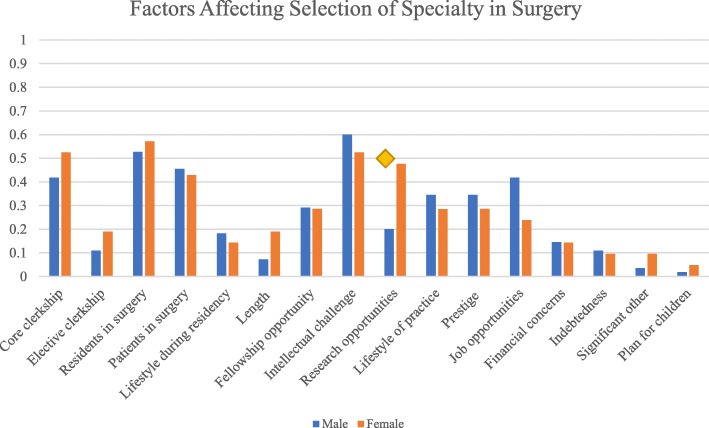
Factors affecting selection of speciality in surgery. Diamond indicates *p* < 0.05

Main factors affecting choice of surgery (Fig. 2) for males included intellectual challenge (60.0%), interaction with residents (52.7%), core clerkship experience (41.8%), and perceived job opportunities (41.8%), whereas, for females, the most common factors for selecting surgery included interaction with residents (57.1%), intellectual challenge (52.4%), and core clerkship experience (52.4%). Out of the respondents that were considering or had already selected their speciality as surgery, females were significantly more likely to join surgery if there were perceived research opportunities compared to males (OR 2.7, *p* = 0.04) (Fig. 2). Otherwise, there were no significant differences between genders in the factors affecting selection of speciality in surgery.

### Factors impinging on selection of surgery as a speciality

**Fig. 3:**
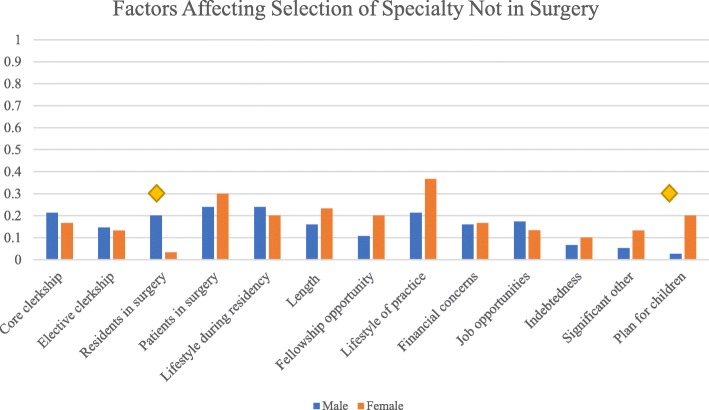
Factors affecting selection of speciality not in surgery. Diamond indicates *p* < 0.05

A wide distribution and variety of factors were cited as reasons for not selecting surgery as a potential speciality (Fig. 3). The most common three reasons for not selecting surgery amongst males included surgical patient population (24.0%), lifestyle during residency (24.0%), and lifestyle of practice (21.3%). For females, the most common factors were lifestyle of practice (36.7%), surgical patient population (30.0%), and length of surgical training (23.3%). The male respondents were more likely than females to report experiences with surgical residents as a significant barrier to selecting surgery (OR 0.26, *p* = 0.03). Female participants were five times more likely than males to select a non-surgical speciality when considering future plans for children (OR 5.1, *p* = 0.01).

## Discussion

Surgically oriented specialities were the top selected fields by junior Rwandan medical students. Almost half of male participants (46.9%) selected surgery as their preferred speciality. Female participants chose obstetrics and gynaecology (29.4%) as their first choice speciality and were twice as likely as compared to their male colleagues to do so (OR 2.14, *p* = 0.05). This preference for a surgical speciality in obstetrics and gynaecology amongst Rwandan females is noteworthy, as non-surgical options were preferred uniformly across other sub-Saharan African countries. In Kenya, a descriptive cross-sectional study showed females being up to eight times more likely to choose non-surgical specialities [8].

Finding a role model was the most common factor involved in selecting any specialisation for both males (73.1%) and females (68.6%). The value of mentorship as an important factor in determining specialisation echoes findings from other African countries. A survey of all medical students in their clinical years in Malawi [10] and a survey of doctors and medical students in Zimbabwe [11] both found that mentorship and positive teaching environments are key elements in guiding female interest and career selection in surgery.

Role models often include residents, and resident interaction was key to shaping career choice for both genders in our study. In many low- and middle-income countries (LMICs), a shortage of personnel and lack of mentorship programs may often limit contact of attending level providers with medical students. For the male participants in Rwanda, interaction with surgeons was a double-edged factor. It was one of the top factors for considering surgery as a speciality (52.7%), but also a reason to choose another field if the interaction had been deemed negative. Males were almost four times more likely than females to report experiences with surgical residents as a barrier to selecting surgery (OR 0.26, *p* = 0.03).

In the United States of America, a study by O’Herrin and colleagues showed that a medical career advisor may help students more purposefully select their speciality based on their unique mix of skills and interests [12]. This kind of career guidance may help broaden students’ career horizon. In Rwanda, there are no known career guidance services offered to students, but may be important for future program development, as it appears that direct experiences with residents and mentors in the department impacted future interest in surgery. While an overarching career guidance service for the medical school may require more intensive resources, concentrated support during the existing clerkship rotations could provide an immediate venue for facilitating mentorship, teaching, and research opportunities.

Other common factors Rwandan female participants cited for influencing speciality choice included fellowship opportunities and intellectual challenge, whether in surgical or non-surgical fields. Additionally, females were twice as likely than their male colleagues to select their speciality when considering research opportunities (OR 1.99, *p* = 0.04). Taken together, these results demonstrate that female Rwandan medical students may value careers with strong academic training and intellectually stimulating opportunities. Male participants were similarly driven to join surgery based on the associated intellectual challenge, but described their core clerkship experience as being influential more often than females did. This drive for intellectual challenge is a finding also frequently found in Asian countries [13]. Previous studies from East Africa instead have also reported that a main reason of choosing surgery as a speciality was the prestige associated with the surgical field [8]. However, this factor was not cited significantly in our study.

Factors that served as barriers to selecting surgery for females were similar in Rwanda to those in many countries around the globe. This gender divide and lack of female surgeons have been well-documented worldwide, with commonly cited reasons for selecting surgery including lifestyle during training, future maternity plans [14, 15], intellectual challenge, perceived prestige, employment options, and lack of mentorship [16, 17]. In Rwanda, female participants were five times more likely than males to cite future plans for children as a factor in their decision process. From our results, it is vital to consider and highlight this finding in the context that females also reported a preference for surgery if research and fellowship opportunities are available. These results may appear contradictory at first glance, but having opportunities to enhance careers with research and facilitating work-life balance are both mechanisms for supporting providers in building their careers. Focusing on these opportunities may encourage female medical students to consider more strongly selecting surgical careers, especially in a context where a preference for surgical specialities may already exist. Many other studies from LMICs have documented similarly that quality of workplace are important factors affecting career selection [18–20].

There are several limitations in this study. First, Rwanda has two schools of medicine, and our study was conducted only in the public medical school. Results from the private school perhaps would have revealed another trend in the factors affecting choice of specialisation. Second, pursuing larger data collection was challenging due to medical students being distributed at various campus locations across the country. The response rate is not ideal as attendance at school is also low for a variety of logistical reasons. Online surveys were also considered, but again, limitations for students accessing the Internet were deemed more obstructive than the multiple sessions of in-person, paper-based methods utilised in this study. Given that the gender distribution of participants (female-to-male ratio of 1:2.5) reflected the average distribution of medical school classes at the University of Rwanda (female-to-male ratio of 1:2.3), this response rate was accepted as appropriate. However, future studies could take advantage of school events with mandatory attendance, such as national examination periods, to improve response rates.

Third, this study was a cross-sectional study, and thus we were not able to assess any changes over time that medical students may experience. A next step for this study is to examine if there is a change between the first and second half of clinical years to evaluate for a trend affecting the choice of specialisation over time. Selection of speciality fluctuates across clinical years, and this study is just an initial cross-sectional study. Improved understanding of the speciality selection process would help leaders determine whether, when, and how to help support these career decisions. Prior studies show that a majority of students indicate interest in a specific speciality and up to 80% change their preference from the time of their medical school entrance examination until the final year of medical school [21]. This study captures junior medical students’ preferences as they complete their core rotations in internal medicine, obstetrics and gynaecology, paediatrics, and surgery. While a few more clinical rotations in other specialities such as anaesthesia and other surgical sub-specialities still remain for exploration for senior Rwandan medical students, the initial exposure to the core specialities influences the early decision of speciality selection. Evaluating early variables influencing students’ decision-making is valuable in understanding the career selection process in Rwanda, and additional studies in our context should strive to include opinions from later medical years as well.

In particular, no students in this study expressed interest in specialising in anaesthesia, a crucial field for being able to provide surgery safely [22]. Rwandan medical students are not necessarily exposed to anaesthesia until their fifth year of study, compared to the four core specialities (internal medicine, surgery, paediatrics, and obstetrics/gynaecology) introduced during their third and fourth year of study. The speciality of anaesthesia in Rwanda is still developing, as technicians were often designated to deliver basic anaesthesia services in common procedures such as caesarean deliveries [23]. It is therefore of recent that the Rwandan Ministry of Health and the anaesthesia department have collaborated to integrate a curriculum for medical students. The capacity to train residents in anaesthesia may affect the number of students considering surgical specialisation to work in the operating room; for now, Rwandan medical students do not seem to be drawn away from surgery toward anaesthesia [23]. Rather, similar concerns about demanding work conditions, insufficient mentorship, and low job opportunity seem to deter Rwandan medical students from ultimately considering anaesthesia too. As such, though early exposure to specialities is important to facilitating greater learning and career-building opportunities, it is important to recognise the changing speciality preferences over time for medical students.

## Conclusion

Strengthening surgical workforce in LMICs is both achievable and central to growing more robust overall health systems. Expanding surgical workforce in Rwanda will require understanding the motivations and factors affecting the process of selecting a speciality. Our study reveals there is a gender divide in career choice amongst junior medical students. Tailoring strategies to match these preferences is important. Creating a mentorship program, fostering positive elective experiences, and providing research opportunities appear to be important factors to help encourage more students to start considering surgery as their specialisation.
